# Directed Thermal Diffusions through Metamaterial Source Illusion with Homogeneous Natural Media

**DOI:** 10.3390/ma11040629

**Published:** 2018-04-19

**Authors:** Guoqiang Xu, Haochun Zhang, Liang Jin, Yan Jin

**Affiliations:** 1 School of Energy Science and Engineering, Harbin institute of Technology, Harbin 150001, China; hitenergyxgq@126.com (G.X.); 13284631700@163.com (L.J.); 2 Center of Applied Space Technology and Microgravity (ZARM), University of Bremen, 28359 Bremen, Germany; Jinyan995072@gmail.com

**Keywords:** thermal diffusion, transformation optics, metamaterial, effective medium theory

## Abstract

Owing to the utilization of transformation optics, many significant research and development achievements have expanded the applications of illusion devices into thermal fields. However, most of the current studies on relevant thermal illusions used to reshape the thermal fields are dependent of certain pre-designed geometric profiles with complicated conductivity configurations. In this paper, we propose a methodology for designing a new class of thermal source illusion devices for achieving directed thermal diffusions with natural homogeneous media. The employments of the space rotations in the linear transformation processes allow the directed thermal diffusions to be independent of the geometric profiles, and the utilization of natural homogeneous media improve the feasibility. Four schemes, with fewer types of homogeneous media filling the functional regions, are demonstrated in transient states. The expected performances are observed in each scheme. The related performance are analyzed by comparing the thermal distribution characteristics and the illusion effectiveness on the measured lines. The findings obtained in this paper see applications in the development of directed diffusions with minimal thermal loss, used in novel “multi-beam” thermal generation, thermal lenses, solar receivers, and waveguide.

## 1. Introduction

The theories of transformation optics (TO) [1] and conformal mapping [2] provide a wide platform for manipulating waves/fluxes under pre-designed methods in various fields, such as electromagnetics [3], optics [4], mechanics [5], acoustics [6], and thermodynamics [7]. Many significant achievements in the above fields, such as the novel invisible cloaks, efficient concentrators, lenses, illusions, etc., have been promoted by employing topological structures which contain form invariances of the governing functions following coordinate transformations. In this case, the tailored topological structures were achieved with near-zero parameters (such as near-zero relative permittivity, relative permeability, and relative thermal conductivity) to reveal the novel manipulative behaviors [8]. Using the pre-designed spatial transformations and the well-established effective medium approximation (EMA) [9], the electromagnetic and optic waves can be precisely controlled and directed through the tailored configurations of relevant material properties. In order to realize the parallel beam shifter and the beam splitter, a modified class of optical transformation was proposed, which employs finite, embedded coordinate transformations to steer the electromagnetic wave [10]. Further studies in embedded optical transformations were carried out, and conversion from the cylindrical waves to plane waves was achieved over a short range, using layered metamaterials [11]. To enhance the feasibility of realizing the tailored material properties, the discrete elements with tailored configurations of relevant material properties were proposed in the fabrication of high-gain lens antennas [12]. Furthermore, directed diffusion, resulting from multi-beam generation [13,14], was both theoretically and experimentally demonstrated. Moreover, camouflaging of the characteristics of the sources by changing the distribution of the electromagnetic fields was also demonstrated. Some attempts of combining the transformation optics and modal analysis have been made, which have motivated the investigation of electromagnetic surface wave devices [15].

Recently, a similar method has been begun to see application in the acoustic field. An acoustic magnifying lens for far-field high-resolution imaging [16] and a source illusion device for flexural Lamb waves [17] were shown to regulate the distribution characteristics of the acoustic field. For the thermal field, some relevant devices have been designed through a generally similar method. A class of thermal metamaterial camouflages [18,19,20], which used to puzzle outside observers, were investigated, which were manufactured by thermally transparent shells attached to customized functional regions. Another type of camouflage [21,22] for creating thermodynamic illusions, i.e., changing arbitrary objects into others, is achieved by reshaping the distributions of heat flux with tailored material configurations. Both of the above kinds of thermal camouflage were utilized to hide the owner characteristics, which should be significant in the existing thermal fields without the tailored material configurations. 

The exploration of source illusions was also opened up recently. The heat flux generated by the central sources was converted from non-parallel patterns into parallel patterns [23]. Furthermore, both 2D and 3D illusion thermal devices [24,25] were investigated to create location camouflages, i.e., transferring the original location characteristics to another area. However, further investigations of such techniques are needed due to the following two concentrations. First, the changing configuration of tailored conductivities was difficult to realize; second, the dependency of the pre-designed profiles restricted the directions of regulating thermal flux diffusions. The classical functions with anisotropic metamaterials [23] are difficult to obtain in practical applications, as the transformed conductivities varied with the positions (including the radii and azimuths). That is, the employed mediums would be changed with the positions. In addition, the classical functions could be efficient once the device profiles were determined, as the entire transformed processes [24,25] were based on the excited characteristic lines. Considering the inconvenience of the classical transformations and the potential applications of directed thermal diffusions, the appropriate transformed methodology that simultaneously avoids singularities and excited characteristic lines, and optimizes the entire process of the linear maps, is of particular importance in achieving highly-efficient arbitrary illusive devices.

In this paper, a class of thermal source illusions is proposed, by considering the space rotations in the linear mapping processes, in order to achieve directed thermal diffusions in arbitrary domains, which are independent of the geometric profiles. In order to adjust the heat flux deflections [26,27,28] with feasible conductivities, homogenous natural media are employed in fabricating the reshaped regions, without limit parameters based on the effective medium theory, which has been widely used in the design of thermal cloaks [29,30,31], concentrators [32], and tunable cells [33,34]. A detailed analysis of the approximate media with relevant fractions, is carried out to select fewer kinds of materials composited in the functional domains. Four schemes with different geometric profiles of isothermal lines, i.e., triangle, square, pentagon, and hexagon, are demonstrated in transient states to validate the proposed derivations of the source illusions. Moreover, the performances are studied by contrasting, based on of the thermal distributions on the measured lines. In addition, the illusion effectiveness of the proposed schemes is investigated as a function of the standard deviations of the temperature deformations. The findings of this work propose a practical method for creating source illusions, through regulating the deflections of heat flux and reshaping distributions of thermal fields. This may greatly benefit the development efforts in thermal energy technologies, such as the thermal lens and directed diffusions with little loss. In addition, the proposed linear mapping methodology with local rotatory positions would also provide a class of novel manipulative functions on plasmonic sensors [35] and design the roadmap of graphene [36].

## 2. Theoretical Derivations of Conductivities Tensors and Geometrical Models

### 2.1. Theoretical Derivation of Conductivities Tensor

**Figure 1:**
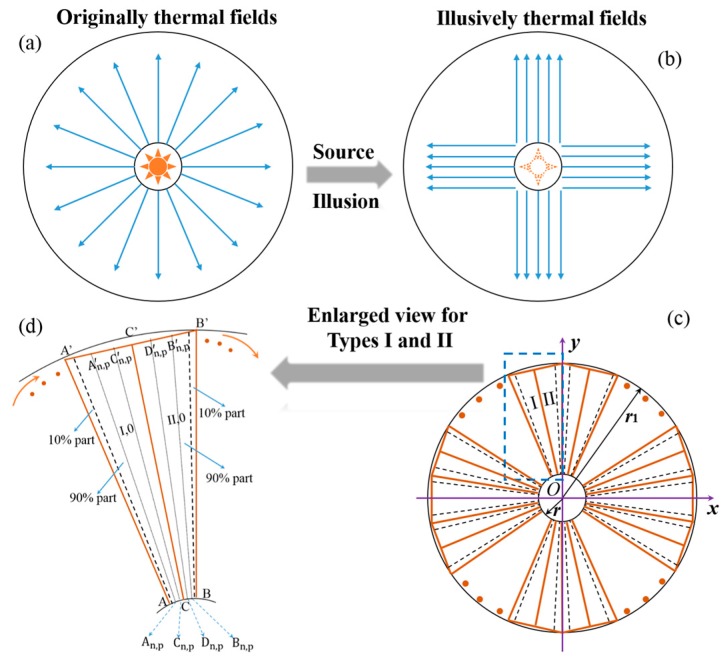
Schematic of the illusive performance and the design of the source illusion device. The blue lines in (**a**,**b**) denote the general heat flux. (**a**) Original thermal fields; (**b**) illusive thermal fields; (**c**) transformation process for the proposed source illusion device; and (**d**) enlarged view of the functional elements labelled by Types I and II.

For realizing the proposed thermal source illusions, which are able to restore the outside thermal flux (temperature distributions) generated by the sources to make thermal illusions with varying characteristics of the existing sources as shown in Figure 1a,b, the schematic of the transformation process is shown in Figure 1c,d.

For simplicity and generality, the entire system is restricted to 2D case, and one vertex of the inscribed polygon of the outer circle is fixed on the line of *x* = 0, i.e., the *y* direction, of the original and transformational domains. Obviously, the proposed source illusions are achieved with topological structures by mapping the inner circles onto the inscribed polygons of the outer circles. To demonstrate the transformation process, the entire system is divided into 2*N* parts through attaching each vertex and midpoint of the inscribed polygons with the origin *O* (0, 0). Furthermore, the separated parts are classified into two types, i.e., the parts with two vertices of (sin((2n−2)π/N), cos((2n−2)π/N)), and ((sin((2n−1)π/N), cos((2n−1)π/N))) are denoted as Type I, in which n ∈ [0, *N*], and the rests are Type II. Note that we adopt the rotation angles between the configurations and *y* directions in this paper. To keep the description of the transformation process simple, the transformations of regions in Type I are taken as an example. It is clear that the linear mapping function [13,14,23] can be employed to respectively map the lines of *OA*_n_ and *OC*_n_ into *OA*_n_′ and *OC*_n_′. The linear mapping can be carried out by validating the relations of the original and transformational domains along the directions of the principle axes. That is, the relevant expansive/compressive coefficients should be solved through the following expressions:

(1) $$\{\begin{array}{c} x^{\prime }=a_{i}x+b_{i}y+c_{i}\\ y^{\prime }=d_{i}x+e_{i}y+f_{i}\\ z^{\prime }=z \end{array}.$$

For the map of arc *A*_n_*C*_n_ to segment *A*_n_*′C*_n_′, it can be directly realized in a simplified way by approximating the length of arc *A*_n_*C*_n_ to segment *A*_n_*C*_n_. However, such a simplification would introduce major errors into the transformations, once the inscribed polygon with fewer edge numbers (*N*), i.e., the length of arc *A*_n_*C*_n_ is not small enough compared with the radius of the inner regions (*r*) [14]. Hence, we further refine the configurations inside the part *A*_n_ *A*_n_*′C*_n_*′ C*_n_ of Type I into m parts through the bisecting angle of *A*_n_*′OC*_n_*′*, in order to reduce the errors due to the simplifications. Each refined configuration is assigned a serial number p_(A~D)_, ranging from 1 to m. That is, the general vertices of an arbitrary element inside Type I can be obtained considering the positions of azimuths:

(2) $$\begin{array}{c} A_{n,p_{A}}\left(r\sin\left(\frac{(2n-2)\pi }{N}+\frac{\left(p_{A}-1\right)\pi }{mN}\right),r\cos\left(\frac{(2n-2)\pi }{N}+\frac{\left(p_{A}-1\right)\pi }{mN}\right)\right);\\ C_{n,p_{C}}\left(r\sin\left(\frac{(2n-2)\pi }{N}+\frac{p_{C}\pi }{mN}\right),r\cos\left(\frac{(2n-2)\pi }{N}+\frac{p_{C}\pi }{mN}\right)\right);\\ A_{{}_{n,p_{A}}}^{\prime }\left(r_{1}\sin\left(\frac{(2n-2)\pi }{N}+\frac{\left(p_{A}-1\right)\pi }{mN}\right),r_{1}\cos\left(\frac{(2n-2)\pi }{N}+\frac{\left(p_{A}-1\right)\pi }{mN}\right)\right);\\ C_{n,p_{C}}^{\prime }\left(r_{1}\cos\frac{\pi }{N}\sin\left(\frac{(2n-2)\pi }{N}+\frac{p_{C}\pi }{mN}\right),r_{1}\cos\frac{\pi }{N}\cos\left(\frac{(2n-2)\pi }{N}+\frac{p_{C}\pi }{mN}\right)\right);\; \end{array}$$

where *r* and *r*_1_ are the radii of the inner and outer circles. n denotes the serial number of each adjacent element inside Type I considering the positions of azimuth, ranging from 0 to *N* − 1. m are the multiples of equal dividing for the central angle and, p_A_ and p_C_ are the serial number of each characteristic component with equal central angle which is counting from the side of *OA*. According to the linear mapping function, the approximate inner triangle $OA_{n,p_{A}}C_{n,p_{C}}$ can be mapped into the outer $OA_{n,p_{A}}^{\prime }C_{n,p_{C}}^{\prime }$ with the following transformation processes upon the linearly expansive processes.

(3) $$\left\{ \begin{array}{l} x_{O}^{\prime }=a_{I,n}x_{O}+b_{I,n}y_{OA_{n,p_{{}_{A}}}}+c_{I,n}\\ x_{A_{n,p_{{}_{A}}}^{\prime }}^{\prime }=a_{I,n}x_{A_{n,p_{{}_{A}}}}+b_{I,n}y_{A_{n,p_{{}_{A}}}C_{n,p_{{}_{C}}}}+c_{I,n}\\ x_{C_{n,p_{{}_{C}}}^{\prime }}^{\prime }=a_{I,n}x_{C_{n,p_{{}_{C}}}}+b_{I,n}y_{C_{n,p_{{}_{C}}}O}+c_{I,n} \end{array}\right.,\left\{ \begin{array}{l} y_{OA_{n,p_{{}_{A}}}^{\prime }}^{\prime }=d_{I,n}x_{O}+e_{I,n}y_{OA_{n,p_{{}_{A}}}}+f_{I,n}\\ y_{A_{n,p_{{}_{A}}}^{\prime }C_{n,p_{{}_{C}}}^{\prime }}^{\prime }=d_{I,n}x_{A_{n,p}}+e_{I,n}y_{A_{n,p_{{}_{A}}}C_{n,p_{{}_{C}}}}+f_{I,n}\\ y_{C_{n,p_{{}_{C}}}^{\prime }O}^{\prime }=d_{I,n}x_{C_{n,p_{{}_{C}}}}+e_{I,n}y_{C_{n,p_{{}_{C}}}O}+f_{I,n} \end{array}\right.$$

Based on the previous studies [16,17], the coefficients of *a*_I,n_, *b*_I,n_, *d*_I,n_, and *e*_I,n_ are the elements of ∂*x′*/∂*x*, ∂*x′*/∂*y*, ∂*y′*/∂*x*, and ∂*y′*/∂*y* in the related Jacobi matrix used for combining such transformation processes. Owing to the 2D domain, the elements of ∂*z′*/∂*x* and ∂*z′*/∂*y* are zero, i.e., *c*_I,n,p_, and *f*_I,n,p_ are independent of the changes in the *z* direction. Hence, the related transformed Jacobi matrix can be derived upon solving the coefficients of *a*_I,n_*, b*_I,n_, *d*_I,n_, and *e*_I,n_. The above equations can be transformed into matrix forms through using the correspondences on the both sides of the simultaneous equations in Equation (3).

(4) $$\left(\begin{array}{c} x_{O}^{\prime }\\ x_{A_{n,p_{{}_{A}}}^{\prime }}^{\prime }\\ x_{C_{n,p_{{}_{C}}}^{\prime }}^{\prime } \end{array}\right)=\left(\begin{array}{ccc} x_{O} & y_{OA_{n,p_{{}_{A}}}} & 1\\ x_{A_{n,p_{{}_{A}}}} & y_{A_{n,p_{{}_{A}}}C_{n,p_{{}_{C}}}} & 1\\ x_{C_{n,p_{{}_{C}}}} & y_{C_{n,p_{{}_{C}}}O} & 1 \end{array}\right)\cdot \left(\begin{array}{c} a_{I,n}\\ b_{I,n}\\ c_{I,n} \end{array}\right),\;\left(\begin{array}{c} y_{OA_{n,p_{{}_{A}}}^{\prime }}^{\prime }\\ y_{A_{n,p_{{}_{A}}}^{\prime }C_{n,p_{{}_{C}}}^{\prime }}^{\prime }\\ y_{C_{n,p_{{}_{C}}}^{\prime }O}^{\prime } \end{array}\right)=\left(\begin{array}{ccc} x_{O} & y_{OA_{n,p_{{}_{A}}}} & 1\\ x_{A_{n,p_{{}_{A}}}} & y_{A_{n,p_{{}_{A}}}C_{n,p_{{}_{C}}}} & 1\\ x_{C_{n,p_{{}_{C}}}} & y_{C_{n,p_{{}_{C}}}O} & 1 \end{array}\right)\cdot \left(\begin{array}{c} d_{I,n}\\ e_{I,n}\\ f_{I,n} \end{array}\right)$$

Obviously, the coefficients of *a*_I,n_, *b*_I,n_, *d*_I,n_, and *e*_I,n_ can be obtained through multiplying the inverse matrices of the corresponding parameters in the original region at the both sides of Equation (4). Considering the positions of characteristic points, the general expansive components in the refined elements of Type I can be achieved as follows.

(5) $$\left(\begin{array}{c} a_{I,n}\\ b_{I,n}\\ c_{I,n} \end{array}\right)={\left(\begin{array}{ccc} x_{O} & y_{OA_{n,p_{{}_{A}}}} & 1\\ x_{A_{n,p_{{}_{A}}}} & y_{A_{n,p_{{}_{A}}}C_{n,p_{{}_{C}}}} & 1\\ x_{C_{n,p_{{}_{C}}}} & y_{C_{n,p_{{}_{C}}}O} & 1 \end{array}\right)}^{-1}\cdot \left(\begin{array}{c} 0\\ r_{1}\sin\left(\frac{(2n-2)\pi }{N}+\frac{\left(p_{A}-1\right)\pi }{mN}\right)\\ r_{1}\cos\frac{\pi }{N}\sin\left(\frac{(2n-2)\pi }{N}+\frac{p_{C}\pi }{mN}\right) \end{array}\right).$$

(6) $$\left(\begin{array}{c} d_{I,n}\\ e_{I,n}\\ f_{I,n} \end{array}\right)={\left(\begin{array}{ccc} x_{O} & y_{OA_{n,p_{{}_{A}}}} & 1\\ x_{A_{n,p_{{}_{A}}}} & y_{A_{n,p_{{}_{A}}}C_{n,p_{{}_{C}}}} & 1\\ x_{C_{n,p_{{}_{C}}}} & y_{C_{n,p_{{}_{C}}}O} & 1 \end{array}\right)}^{-1}\cdot \left(\begin{array}{c} 0\\ r_{1}\cos\left(\frac{(2n-2)\pi }{N}+\frac{\left(p_{A}-1\right)\pi }{mN}\right)\\ r_{1}\cos\frac{\pi }{N}\cos\left(\frac{(2n-2)\pi }{N}+\frac{p_{C}\pi }{mN}\right) \end{array}\right).$$

For the elements in the parts of Type II, the conductivities can be obtained using the similar mapping function. The general positions of the vertices inside the elements of Type II are presented as follows:

(7) $$\begin{array}{l} B_{n,p_{B}}\left(r\sin\left(\frac{2n\pi }{N}-\frac{\left(p_{B}-1\right)\pi }{mN}\right),r\cos\left(\frac{2n\pi }{N}-\frac{\left(p_{B}-1\right)\pi }{mN}\right)\right);\\ D_{n,p_{D}}\left(r\sin\left(\frac{2n\pi }{N}-\frac{p_{D}\pi }{mN}\right),r\cos\left(\frac{2n\pi }{N}-\frac{p_{D}\pi }{mN}\right)\right);\\ B_{{}_{n,p_{B}}}^{\prime }\left(r_{1}\sin\left(\frac{2n\pi }{N}-\frac{\left(p_{B}-1\right)\pi }{mN}\right),r_{1}\cos\left(\frac{2n\pi }{N}-\frac{\left(p_{B}-1\right)\pi }{mN}\right)\right);\\ D_{n,p_{D}}^{\prime }\left(r_{1}\cos\frac{\pi }{N}\sin\left(\frac{2n\pi }{N}-\frac{p_{D}\pi }{mN}\right),r_{1}\cos\frac{\pi }{N}\cos\left(\frac{2n\pi }{N}-\frac{p_{D}\pi }{mN}\right)\right);\; \end{array}$$

In Equation (7), p_B_ and p_D_ are the corresponding serial number of each characteristic component counting from the side of *OB*. Considering the mapping relations and the characteristic points, the expansive coefficients of the elements inside Type II can be derived by taking Equation (7) into Equation (1). Hence, the general expansive coefficients of Type II can be obtained.

(8) $$\left(\begin{array}{c} a_{\mathrm{II},n}\\ b_{\mathrm{II},n}\\ c_{\mathrm{II},n} \end{array}\right)={\left(\begin{array}{ccc} x_{O} & y_{OB_{n,p_{B}}} & 1\\ x_{B_{n,p_{B}}} & y_{B_{n,p}D_{n,p_{D}}} & 1\\ x_{B_{n,p_{B}}} & y_{Dn,p_{D}O} & 1 \end{array}\right)}^{-1}\cdot \left(\begin{array}{c} 0\\ r_{1}\sin\left(\frac{2n\pi }{N}-\frac{\left(p_{B}-1\right)\pi }{mN}\right)\\ r_{1}\cos\frac{\pi }{N}\sin\left(\frac{2n\pi }{N}-\frac{p_{D}\pi }{mN}\right) \end{array}\right).$$

(9) $$\left(\begin{array}{c} d_{\mathrm{II},n}\\ e_{\mathrm{II},n}\\ f_{\mathrm{II},n} \end{array}\right)={\left(\begin{array}{ccc} x_{O} & y_{OB_{n,p_{B}}} & 1\\ x_{B_{n,p_{B}}} & y_{B_{n,p}D_{n,p_{D}}} & 1\\ x_{B_{n,p_{B}}} & y_{Dn,p_{D}O} & 1 \end{array}\right)}^{-1}\cdot \left(\begin{array}{c} 0\\ r_{1}\cos\left(\frac{2n\pi }{N}-\frac{\left(p_{B}-1\right)\pi }{mN}\right)\\ r_{1}\cos\frac{\pi }{N}\cos\left(\frac{2n\pi }{N}-\frac{p_{D}\pi }{mN}\right) \end{array}\right).$$

Taking Equations (5), (6), (8), and (9) together, the Jacobi matrix for realizing such 2D expansive actions are expressed as:

(10) $$J=\frac{\partial \left(x^{\prime },y^{\prime }\right)}{\partial \left(x,y\right)}=\left(\begin{array}{cc} \frac{\partial x^{\prime }}{\partial x} & \frac{\partial x^{\prime }}{\partial y}\\ \frac{\partial y^{\prime }}{\partial x} & \frac{\partial y^{\prime }}{\partial y} \end{array}\right)=\left(\begin{array}{cc} a_{i,n} & b_{i,n}\\ d_{i,n} & e_{i,n} \end{array}\right)\cdot$$

where *i* denotes the types of the functional regions, i.e., Type I or II. Using the above Jacobi matrix, the conductivity components of each element, for achieving the proposed source illusions in the expansive regions, are obtained through solving the expressions as: $\kappa^{\prime }\;=\;\frac{J\cdot \kappa \cdot J^{\prime }}{\det\left(J\right)}$ with det (*J*) = *a_i_*_,n_·*e_i_*_,n_ − *b_i_*_,n_·*f_i_*_,n_, in which *κ* denotes the original conductivity between the inner and outer circles. Hence, the conductivity components can be expressed as follows:

(11) $$\kappa_{xx}^{\prime }=\frac{\left(a_{i,n}^{2}+b_{i,n}^{2}\right)}{\det(J)}\kappa .$$

(12) $$\kappa_{xy}^{\prime }=\kappa_{yx}^{\prime }=\frac{\left(a_{i}{}_{,n}d_{i,n}+b_{i,n}e_{i,n}\right)}{\det(J)}\kappa .$$

(13) $$\kappa_{yy}^{\prime }=\frac{\left(d_{i,n}^{2}+e_{i,n}^{2}\right)}{\det(J)}\kappa .$$

The components of the conductivities of Types I and II for the source illusions can be derived through Equations (11)–(13), by separately considering the transformations in Equations (5), (6), (8), and (9). Note that the values det (*J*) both in Types I and II are $\frac{r_{1}^{2}}{r^{2}}\cos\left(\frac{\pi }{N}\right)$, owing to the linear mapping functions along the principle axes of the relevant curves. Hence, the components of conductivities of the proposed source illusions are independent of the range of the transformed regions. That is, the thermal illusions would theoretically be observed outside the outer circles, once the conductivity components in the characteristic direction are in accordance with the values calculated by Equations (11)–(13), without considering the sizes of the inner and outer circles.

For the sake of comparisons with those methods “isotropic cloaking formalism” and “anisotropic cloaking formalism” [37,38] employed in manipulating surface waves, the proposed method with local rotational and radial components can be used to achieve varied thermal behaviors by employing different geometric components. Owing to the linear maps between characteristic lines, the rotationally-symmetric profiles are not required, and the limit parameters can be significantly reduced.

### 2.2. Selections of Materials and Geometric Profiles of Thermal Source Illusions

The theoretically general components of the relevant thermal conductivity tensors of the elements inside Types I and II of the proposed source illusions are presented above. The next work is proposing the method for establishing such devices. As [26] pointed out, the relative conductivities for such composited systems, considering the deflections of heat flux [26,27,28], i.e., rotations of the local coordinate systems, could be obtained through alternative configurations of layered materials. Hence, the relative conductivities along the principle axes for functional regions of the source illusions can be deduced based on the effective medium theory.

(14) $$\left(\begin{array}{cc} \kappa_{x}^{\prime } & 0\\ 0 & \kappa_{y}^{\prime } \end{array}\right)=R(\theta )\cdot \left(\begin{array}{cc} \frac{r^{2}\left(a_{i,n}^{2}+b_{i,n}^{2}\right)}{r_{1}^{2}\cos\left(\frac{\pi }{N}\right)}\kappa & \frac{r^{2}\left(a_{i}{}_{,n}d_{i,n}+b_{i,n}e_{i,n}\right)}{r_{1}^{2}\cos\left(\frac{\pi }{N}\right)}\kappa \\ \frac{r^{2}\left(a_{i}{}_{,n}d_{i,n}+b_{i,n}e_{i,n}\right)}{r_{1}^{2}\cos\left(\frac{\pi }{N}\right)}\kappa & \frac{r^{2}\left(d_{i,n}^{2}+e_{i,n}^{2}\right)}{r_{1}^{2}\cos\left(\frac{\pi }{N}\right)}\kappa \end{array}\right)\cdot R(\theta ){}^{\prime }$$

In Equation (14), *θ* denotes relative rotation angle between the local configurations of the layered materials and corresponding principle axes (*y* axes). *R*(*θ*) and *R*(*θ*)′ are the unimodular rotation matrices. Furthermore, the relative conductivities along the principle axes can be obtained based on effective medium theory with the fact of $\theta =\frac{\pi }{2}-\frac{1}{2}\arctan\left(\frac{2\left(a_{i,n}d_{i,n}+b_{i,n}e_{i,n}\right)}{a_{i,n}^{2}+b_{i,n}^{2}-d_{i,n}^{2}-e_{i,n}^{2}}\right)$. Considering the findings from studies in bending heat flux with layered material configurations, the requirements of the relative conductivity components along the principle axes could be achieved by alternative configurations of negative and positive layers, with certain area fractions [29,32,34].

(15) $$\kappa_{x}^{\prime }=\frac{\kappa_{P}\cdot \kappa_{N}}{\gamma_{P,l}\cdot \kappa_{N}+\gamma_{N,l}\cdot \kappa_{P}}$$

(16) $$\kappa_{y}^{\prime }=\gamma_{P,l}\cdot \kappa_{P}+\gamma_{N,l}\cdot \kappa_{N}$$

where, *κ*_P_ and *κ*_N_ are the relative conductivities for the positive and negative layers. *γ*_P,l_ and *γ*_N,l_ denote the composited fractions of the two layers in certain regions and the sum of *γ*_P,l_ and *γ*_N,l_ is 1.

In order to validate the derivations and demonstrate the source illusions, four schemes with varying profiles of the inscribed polygons, including triangle (*N* = 3), square (*N* = 4), pentagon (*N* = 5), and hexagon (*N* = 6), are proposed. As the illusion performances are independent of the physical dimensions, i.e., the radii of the inner circular spaces (*r*) where the thermal source located, and the function regions between the inner and outer circles (*r*_1_), the radii of the outer (inscribed polygons) and inner circles are set as 0.1 m and 0.02 m, i.e., *r*_1_ = 0.1 m and *r* = 0.02 m. The entire systems are placed at the center of the square plates with dimensions of 400 mm × 400 mm, filled with nickel steel (50% Ni) with a thermal conductivity of *κ* = 19.6 W∙m^−1^∙K^−1^. In addition, the inner circles are used for diffusing the thermal flux generated by the sources. Hence, the nickel steel (50% Ni) is also employed to fabricate the inner circular regions as it has small effect on the restoring function. For the parts between the outer circle and corresponding inscribed polygons, nickel steel is also employed, as the related spaces are unchanged during the transformation process. Considering the characteristics of the designed distribution of thermal fields under related polygonal profiles, each area inside Types I and II of all the schemes is divided into two parts: 10% portions of the area at the positions of the geometric deformations are considered as one part, while the remaining 90% are another. The 10% separations are beneficial to restrict the regions with extreme conductivities in relatively small areas. In addition, this also helps by reducing the limit parameters at the geometric deformations, resulting in better approximate mapping. The following considerations are to select the appropriate positive and negative layers for creating the 10% and 90% parts of the functional regions. For the 10% part in each type, p_A(B)_ and p_C(D)_ should be 1, and the values of m should be 22.4, 15, 12.8, and 11.8 for the triangle, square, pentagon, and hexagon schemes, respectively. In the 10% part of Type I, the relative conductivities for the triangle and square schemes are far larger than natural positive mediums, and the relative conductivities for the pentagon and hexagon are 291.52 and 199.75 W∙m^−1^∙K^−1^ with nearly 100% fractions. In order to satisfy the requirements of the conductivities for the 10% parts in Type I, the pure copper with a conductivity of 398 W∙m^−1^∙K^−1^ is selected for the triangle, square, and pentagon schemes. Aluminum alloy (6063) with a conductivity of 201 W∙m^−1^∙K^−1^ is employed in the hexagon scheme. Furthermore, the adjacent 10% parts of the Types II are all filled with polydimethylsiloxane (PDMS) to create essential anisotropies coupled with those former 10% parts at the geometric deformations (near the vertices).

**Figure 2:**
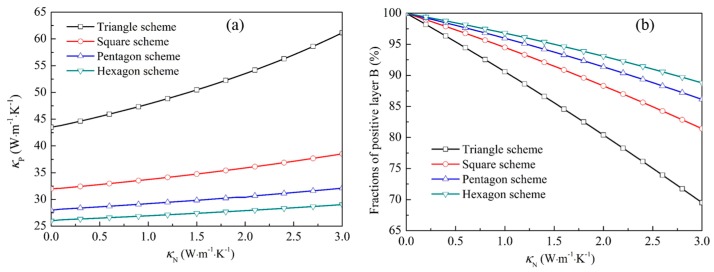
(**a**) The thermal conductivities of the positive layers B; and (**b**) the related fractions of the positive layers B.

For the 90% part in each type, p_A(B)_ is 2, and the values of m and p_C(D)_ should also be 22.4, 15, 12.8 and 11.8 for the triangle, square, pentagon, and hexagon schemes, respectively. Taking the above newly defined points into Equations (5), (6) and (8), (9), the corresponding conductivity components and related fractions of the positive and negative layers can be achieved by Equations (14)–(16). Here, the negative and positive layers are respectively defined as layers A and B. Owing to the symmetries of the distribution of the functional regions, the configurations of the conductivities in the positive and negative layers are uniform. The desired conductivities of positive layers B and related fractions for the 90% part of Types I and II are determined as a function of the conductivities of the negative layers A, as shown in Figure 2.

It can be seen that the conductivities of layers B increased with those of the negative layers. Meanwhile, the composited fractions of layers B simultaneously reduced in order to satisfy the demands calculated by Equations (15) and (16) based on the effective medium theory [26]. In order to obtain the appropriate conductivities in the negative and positive layers for realizing such source illusions, the polydimethylsiloxane (PDMS) with a conductivity of 0.15 W∙m^−1^∙K^−1^ is employed to completely fill in the layers A, owing to its low conductivity and preference of industry. For the conductivities of layers B, the values for the proposed triangle, square, pentagon, and hexagon schemes should be 44.015, 32.172, 28.233, and 26.232 W∙m^−1^∙K^−1^, respectively. Considering the previous studies [29,32,34], the conductivities inside layers B could be achieved by alternately arranging negative and positive mediums, owing to the approximate values calculated by Equation (16) and $\kappa_{P,l}=\alpha_{P,\;m}\kappa_{P,m}+\alpha_{N,m}\kappa_{N,m}$ (where *α*_P,m_ and *α*_N,m_ denote the area fractions of the positive and negative media composited in layers B, and *κ*_P,m_ and *κ*_N,m_ are the corresponding conductivities for the positive and negative media). In order to design the schemes with fewer kinds of media, the negative media composited in layers B are also PDMS. Hence, the total fractions of employed mediums inside this part (90% part) can be obtained.

(17) $$\beta_{P,T}=\alpha_{P,m}\cdot \gamma_{P,l},$$

(18) $$\beta_{N,T}=\gamma_{N,l}+\alpha_{N,m}\cdot \gamma_{P,l}.$$

In Equations (17) and (18), *α*_P,m_ and *α*_N,m_ are the fractions of the positive and PDMS composited in the each positive layer. *β*_P,T_ and *β*_N,T_ denote the total fractions of the positive and negative media (PDMS) employed in each 90% part of all types, respectively. Furthermore, the more balanced configurations of the positive media and PDMS, the optimal expected performances would occur, owing to the higher anisotropies inside the functional regions.

**Figure 3:**
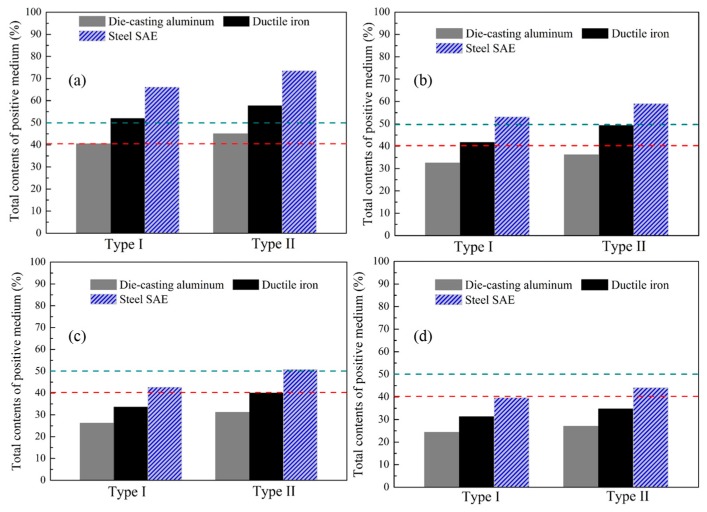
Total contents of the contents of the potentially positive candidates for the proposed schemes. (**a**) Triangle scheme; (**b**) square scheme; (**c**) pentagon scheme; and (**d**) hexagon scheme.

Considering the 90% part of the separated structures discussed above, the appropriate positive media, whose total composited fractions approach 40% in Types I and 50% in Types II, should be adopted for all the proposed schemes. Hence, die-casting aluminum A380 with a conductivity of 96.2 W∙m^−1^∙K^−1^, ductile iron with a conductivity of 75 W∙m^−1^∙K^−1^, and steel SAE 1010 with a conductivity of 59 W∙m^−1^∙K^−1^ are selected as the potential candidates for the proposed schemes. The total contents of the candidates in each scheme are illustrated in Figure 3. In order to employ fewer kinds of media in each scheme, only one positive medium, which approximately and simultaneously satisfied the fractional demands in Types I and II. Hence, die-casting aluminum A380 is selected for the triangle scheme with total fractions of 40.54% and 45.05% for Types I and II. Ductile iron is used for Types I and II of the square scheme with corresponding total fractions of 41.78% and 49.42%. Moreover, steel SAE is employed both in the pentagon and hexagon schemes, due to its most approximate fractions to the demands, i.e., 42.70% and 50.83% for Types I and II of the pentagon scheme, and 39.71% and 44.12% for Types I and II of the hexagon scheme.

**Figure 4:**
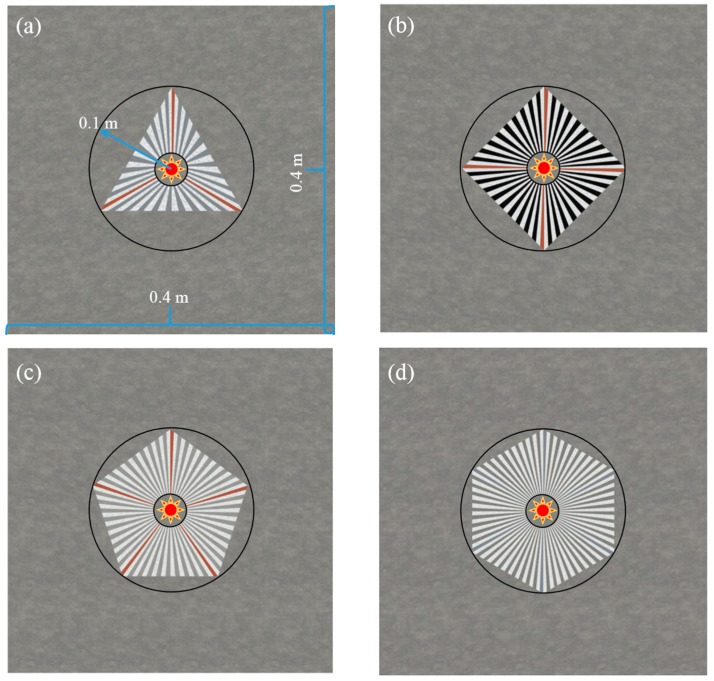
Geometrical models for the proposed thermal source illusive schemes with varying inscribed polygons. (**a**) The triangle scheme; (**b**) the square scheme; (**c**) the pentagon scheme; and (**d**) the hexagon scheme (see the section in the Appendix for the summary for the employed mediums and related fractions).

Considering the thermal expanding effects of the functional regions (Types I and II), the wedge cell structures are employed for fabricating the positive and negative medium layers. Taking into account the selected media and related fractions, both in the 10% and 90% parts of Types I, one pure positive wedge layer (copper/aluminum alloy) is ordinarily employed in the 10% part for the proposed schemes. Five PDMS wedge layers and four positive medium (die-casting aluminum A380, ductile iron, and steel SAE 1010) wedge cells are embedded into the 90% part, as part of the total fractions. In analogy to Types II, one pure PDMS layer is embedded in the 10% part. Five positive medium layers and four PDMS wedge layers are employed in the rest regions with homologous fractions. The geometrical models for the proposed source illusion schemes are illustrated in Figure 4. Different from the previous contributions on electromagnetic meta-devices [39,40], the proposed linear maps methodology has been expanded into thermal fields with the considerations of local radial and angular components. Considering the general electromagnetic applications [41,42], it is believed that the designed illusive schemes based on the proposed methodology could open up an avenue to utilize the excited heat source efficiently in practical applications of the thermal field.

In order to validate and demonstrate the proposed schemes, numerical simulations based on the finite volume method are employed with ANSYS Fluent to solve the energy differential equation by adopting the second-order difference scheme. The boundaries of the calculated domains are thermally insulated, and the point sources are located at the center of the entire systems with constant temperatures *T*_s_ = 373 K. Furthermore, a scheme of pure nickel steel (50% Ni) plate with dimensions of 400 mm × 400 mm is established to make fair contrasts with the proposed schemes.

## 3. Demonstration of the Proposed Schemes and Discussions

**Figure 5:**
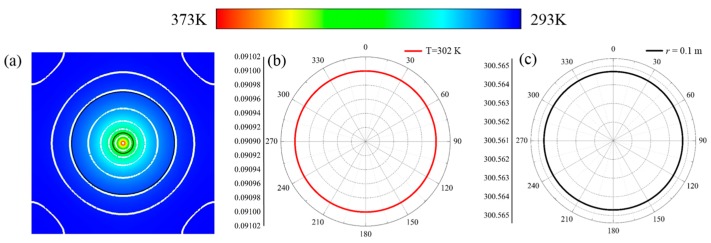
Distribution characteristics of the thermal fields for the contrast bare plate scheme. (**a**) Temperature distributions of the entire bare plate scheme; (**b**) distribution characteristics on the isothermal line of *T* = 302 K; and (**c**) temperature distributions on the measured line of *r* = 0.1 m.

Based on the transformation derivations and relevant thermal expanding effects, the temperature distributions of the contrasting pure nickel steel plate and the proposed source illusion schemes are obtained at t = 1000 s. Figure 5 illustrates the temperature distributions of the pure nickel steel plate. This indicates that the thermal energy generated by the point source diffused uniformly and forced the isothermal lines in the calculated domains, approaching circles as shown in Figure 5a, owing to the isotropies in the contrast scheme. In order to describe the characteristics of the temperature distributions and to make striking contrasts for between the following source illusion schemes, two characteristic lines, including the isothermal line of *T* = 302 K and the measured line of *r* = 0.1 m, are selected and illustrated in polar coordinates. As shown in Figure 5b, the *r* direction was defined as the radii of the locations of *T* = 302 K. It can be seen that the isothermal line formed in the circle profile, as the radii on the isothermal line of *T* = 302 K were 0.091 m, which were in accordance with that shown in Figure 5a. The temperature distributions on the line of *r* = 0.1 m were shown in Figure 5c. Obviously, the temperatures on the line of *r* = 0.1 m were all 300.6 K. That is, the thermal energy diffused uniformly along all the directions inside the system.

### 3.1. Temperature Distributions of the Proposed Thermal Source Illusions

**Figure 6:**
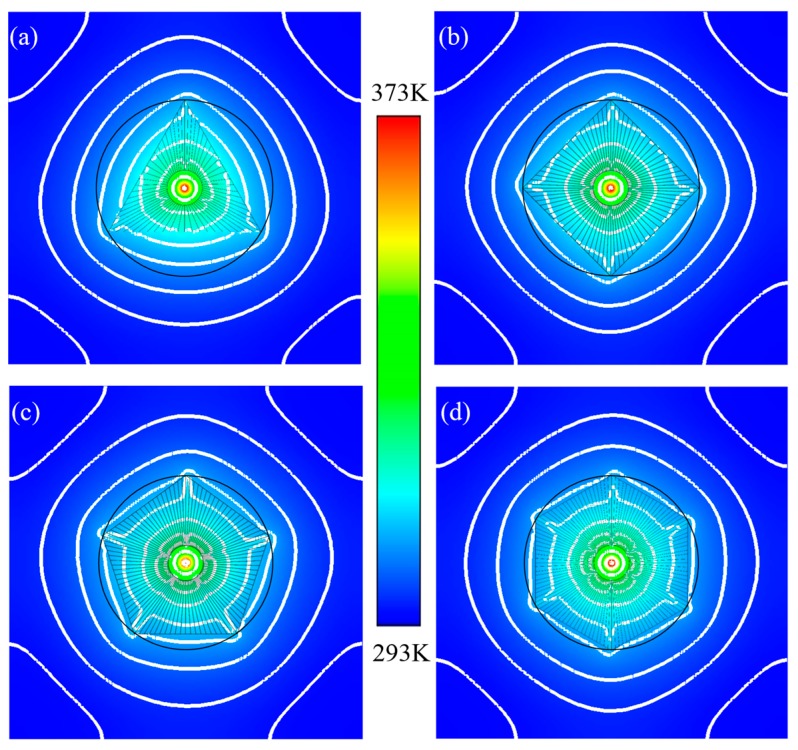
Temperature distributions for proposed schemes at t = 1000 s. (**a**) The triangle scheme; (**b**) the square scheme; (**c**) the pentagon scheme; and (**d**) the hexagon scheme.

The temperature distributions of the proposed thermal source illusions at t = 1000 s are shown in Figure 6. The temperature distributions outside the inscribed polygonal regions were shaped according to the pre-designed polygonal profiles. That is, the outer thermal fields were reshaped into designed polygons, and all the illusive performances were significant.

For the triangle scheme, the thermal energy diffused uniformly inside the inner circle region, i.e., the distributions of isothermal lines were shaped in circular profiles, owing to the inside isotropic material configurations and the point sources. Moreover, the distributions of the thermal energy were adjusted in the functional region, where the Types I and II were located, owing to the thermal expanding effects inside the functional regions. The isothermal lines were manipulated by the function elements of Types I and II, which forced non-uniform diffusions of the thermal energy, due to the alternating configurations of the positive and negative media.

With the outwards expansive diffusion, the thermal energies, which were located at the central regions corresponding to the outer boundaries, diffused into the outer domains of the triangle functional region faster than those located at the geometric deformations (near the vertices). That is, the outward diffusions of thermal energies were enhanced, given the reduced distances from the points on the boundaries to the origin *O* (0, 0). Furthermore, the refined configurations of the 10% fractional part at the geometric deformations promoted the thermal diffusions in the wedge elements approaching the vertices, owing to the high conductivities of the selected media. Hence, the profiles of the isothermal lines varied violently near the vertices of the outer triangle, which optimized distributions of the thermal fields outside the functional regions (outer circle) and reshaped profiles of the isothermal lines into triangular distributions. Thus, thermal source illusions occurred in the outer domains, which hid the original characteristics of the sources inside the central circle regions, and changed those into the pre-designed thermal distribution characteristics. Similar to the other inscribed polygon schemes, the distributions of the thermal fields were reshaped, by virtue of the functional wedge elements inside the inscribed polygons. Moreover, the profiles of the isothermal lines, which approached to the polygon boundaries, would better approximate the shapes of the inscribed polygons with the increasing side numbers of the polygons. That is, the more similar temperatures outside the outer circles were observed along the corresponding profiles of the inscribed polygons, owing to the reduced areas of the domains between the outer circles and the inscribed polygons.

### 3.2. Characteristics of Temperature Distribution

**Figure 7:**
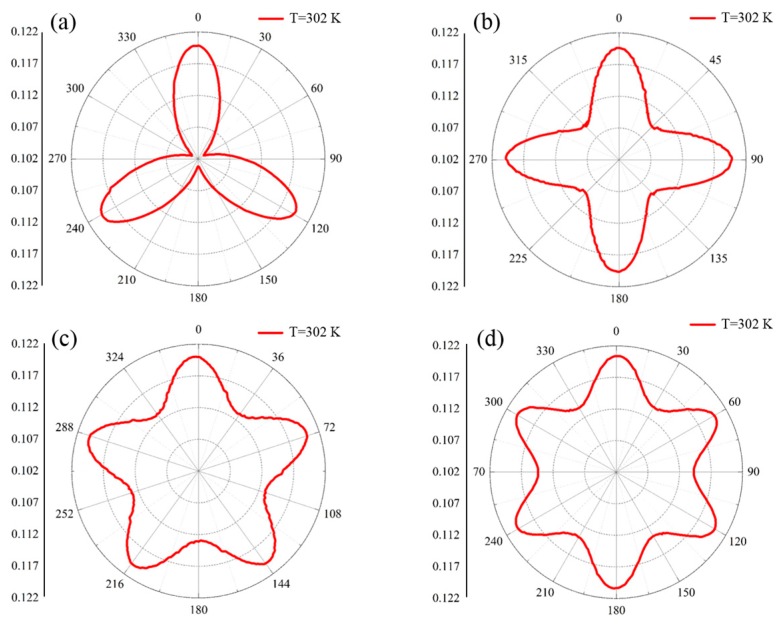
Distribution characteristics on the isothermal lines of *T* = 302 K for proposed schemes. (**a**) The triangle scheme; (**b**) the square scheme; (**c**) the pentagon scheme; and (**d**) the hexagon scheme.

In order to further investigate the characteristics of the temperature distributions of the proposed schemes, the isothermal lines of *T* = 302 K and the measured line of *r* = 0.1 m were also selected. The characteristics of the temperature distributions of the isothermal lines of *T* = 302 K for the proposed schemes are illustrated in Figure 7. Obviously, the distribution characteristics of the thermal fields changed violently compared with that of the bare plate scheme shown in Figure 4b. As illustrated in Figure 6a, the radii of the locations of *T* = 302 K drastically fluctuated in the ranges of *θ* = 0°–120°, 120°–240°, and 240°–360°, and centered on *θ* = 60°, 180°, and 300°, i.e., the ranges of the changing directions corresponded to the boundaries of the inscribed triangle. The radii of the locations rapidly decreased in the range of 0°–60° and then increased in the range of 60°–120°, due to the thermal flux manipulations of the functional elements inside the inscribed polygons. This indicates that the distributions of thermal fields were regulated following the profile of the pre-designed triangle, as the radii of the locations of *T* = 302 K varied gradually following the trends of the geometrical structure corresponding to the azimuths. In addition, the maximums of the locational radii appeared at the azimuths of *θ* = 0°, 120°, and 240°, while the minimums were observed at *θ* = 60°, 180°, and 300°. Thus, the profiles of the isothermal line of *T* = 302 K were approximately shaped in the triangle profile upon the above findings. However, the changing trends of the radii approached the azimuths of *θ* = 0°, 120°, and 240° were larger than those of the boundaries of the inscribed triangle, which followed the function of $r_{l}=(r_{1}\cos\frac{\pi }{N})/\sin\left(\theta +\frac{\pi }{2N}\right)$, where *r*_l_ denotes the radius on the boundary of the inscribed triangle. That is, the nature of thermal diffusions led to the local expansion of energy distributions with pure nickel steel inside the domains between the inscribed triangle and the outer circle. Similar to the other schemes, the maximums occurred, where the azimuths approached the vertices of the inscribed polygons, and the minimums were observed at the azimuths where the symmetry axes were located, i.e., *θ* = 45°+nπ/4, 36° + nπ/5, and 30° + nπ/6 for the square, pentagon, and hexagon schemes (n ranges from 0 to *N* − 1). Furthermore, the inner regions inside the isothermal lines were enlarged with the increasing side counts of the inscribed polygons, which forced boundaries to approach the outer circles caused by the decreasing central angles. Meanwhile, the above phenomena also meant that the thermal energy distributions were expanded by the functional elements compared with the contrast scheme shown in Figure 5b, i.e., the minimum radii of the isothermal lines of *T* = 302 K were larger than those in Figure 5b.

**Figure 8:**
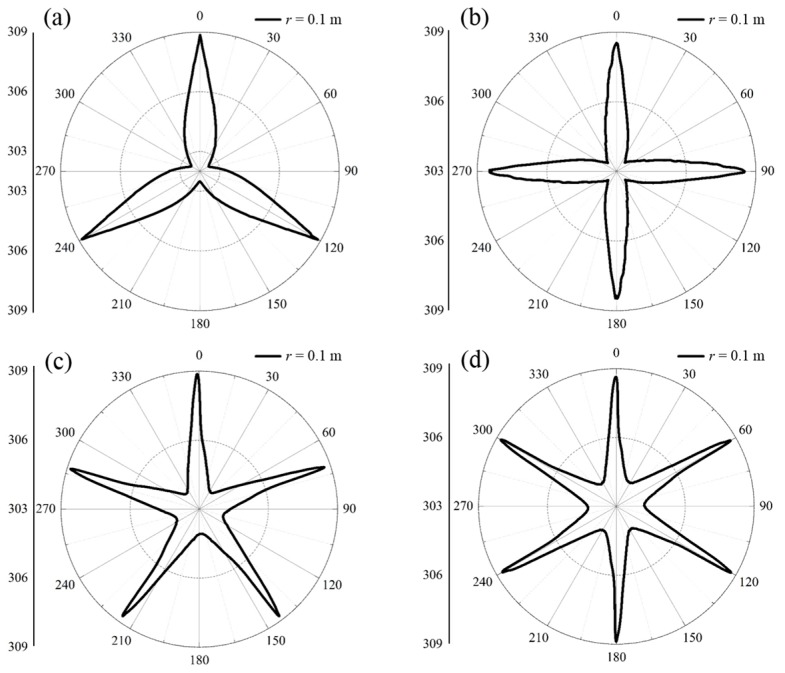
Distribution characteristics on the measured lines of *r* = 0.1 m for proposed schemes. (**a**) The triangle scheme; (**b**) the square scheme; (**c**) the pentagon scheme; and (**d**) the hexagon scheme.

Figure 8 provides the temperature distributions of the measured lines of *r* = 0.1 m (the outer circles) for the proposed schemes. It can be seen that the highest temperatures were observed at the positions of the geometric deformations of the inscribed polygons for all the proposed schemes, i.e., the vertices of the inscribed polygons located at the azimuths of *θ* = 2nπ/*N*. The lowest temperatures appeared at the azimuths of *θ* = (n + 1)·π/*N* in the proposed schemes. In addition, the lowest temperatures on the measured lines increased with the increasing side numbers. That is, the distributions of thermal energies were expanded outwards, which were in accordance with those illustrated in Figure 7. Furthermore, the temperatures first rapidly decreased and then increased at the azimuths ranging from 2nπ/*N* to 2(n + 1)·π/*N*, and centered on the azimuths of *θ* = (n + 1)·π/*N*, where the lowest temperatures were observed. Moreover, the changing trends of the temperatures increased with the increasing side counts, owing to the decreasing central angles of the inscribed polygons. In general, the temperatures near the vertices were significantly higher and the associated isothermal lines were forced to run parallel to the boundaries of the inscribed polygons, benefiting from the optimal conductivities in the 10% parts of the functional domains and the directed diffusions processes inside the 90% parts. Hence, the profiles of the isothermal lines outside the outer circle domains were approximately shaped in pre-designed profiles, and performed illusions for the actual sources through regulating the outside distributions of the thermal fields.

To further examine the illusion effectiveness of the proposed schemes, another seven measured lines of *r* = 0.04, 0.06, 0.08, 0.12, 0.14, 0.16, and 0.18 m were selected to calculate the temperature deformations [30] between the proposed schemes and the contrast bare plate, i.e., *T*_D_ = |*T*_sc_ − *T*_pl_|, where *T*_sc_ and *T*_pl_ are the temperatures on the measured lines of the proposed schemes and bare plate, respectively. The standard deviations of the temperature deformations on the measured lines, used to express the effectiveness, can be written as:

(19) $$\sigma =\sqrt{\frac{1}{N_{m}}\sum_{i=1}^{N_{m}}{\left(T_{D}-\bar{T_{D}}\right)}^{2}}$$

**Figure 9:**
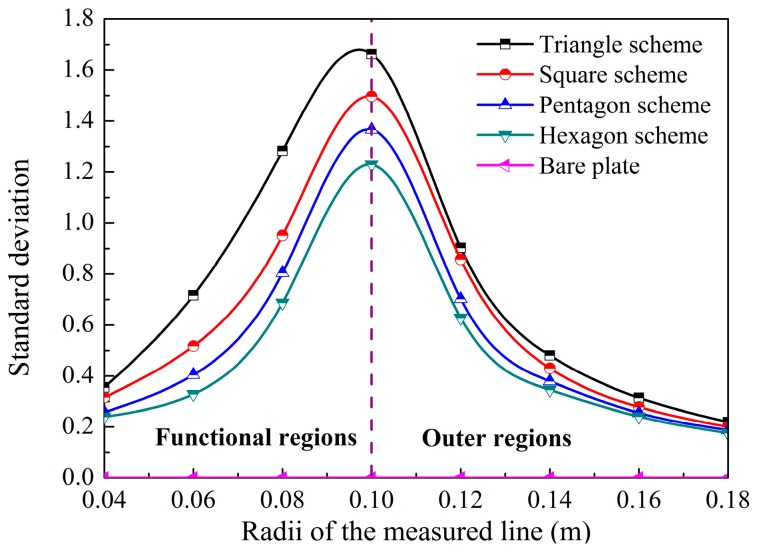
Standard deviations of the temperature deformations for the proposed schemes as a function of the radii of the additional measured lines.

In Equation (19), *N*_m_ is the numbers of the measured points on related measured lines. The standard deviations of the temperature deformations on the measured lines are shown in Figure 9. Note that the larger standard deviations indicate higher discreteness of the temperature deformations between observed values and their averages. Consequently, larger deformations would appear on corresponding azimuths of the proposed schemes, owing to the symmetries illustrated in Figure 5. Hence, more significant performances of illusions would be observed. As Figure 9 illustrates, the standard deviation on each measured line was 0 for the contrast bare plate, which contributed to shape the profiles of the temperature distributions as circles. For the proposed schemes, the standard deviations on each measured line increased with decreasing side numbers, which meant that the more significant illusions were achieved in the schemes with fewer diffusion elements inside the functional regions. For the independent schemes, the trends of the standard deviations as a function of the radii of the measured lines were similar, i.e., first increased in the functional regions and then decreased in the outer regions. In addition, the more approximate deformations would be observed in the schemes with more diffusion elements, i.e., more side counts. Hence, the more approximate temperature distributions were observed on the related azimuths with fewer side counts, due to the reduced central angles, which were in accordance with those shown in Figure 5 and validated in the electromagnetic field [14]. For the thermal diffusions in the outer spaces, the standard deviations decreased as the radii increased, due to the natural characteristics of the diffusions inside the isotropic medium of the outer spaces with thermal losses. Furthermore, the thermal fluxes were able to transfer efficiently, which could be orthogonal to the pre-designed boundaries of the inscribed polygons in the outer domains, once the media inside the outer spaces were compiled following certain conditions.

## 4. Conclusions

In general, a methodology for generally achieving directed thermal diffusions with thermal source illusions is proposed by setting several functional elements with the linear mapping function. The employment of space rotations can promote the fabrications of arbitrary thermal illusions in global transformation domains considering the geometric deformations on any azimuth. Note that such a methodology could perform the best results, once the length of arc of is small enough compared with the radius in the original domain, due to the geometric approximations in the design process. The details of the appropriate natural material selections with relevant fractions in varied illusion schemes have been investigated based on the effective medium theory. Fewer kinds of natural homogenous media for each proposed scheme, including the triangular, square, pentagonal, and hexagonal, have been employed to avoid the extreme parameters and improve the feasibility. The expected illusive performances and noticeably reshaped profiles of the isothermal lines have been observed in transient states. That is, the directed thermal diffusions through homogenous medium devices were validated, owing to the thermal-expansive effects inside the functional types. Furthermore, the performances and effectiveness of the source illusions have been demonstrated by characteristics of the measured lines and the standard deviations of the temperature deformations. The contrasts indicated that more approximate profiles to inscribed polygons of the isothermal lines, i.e., the better directed thermal diffusions, would be observed with more polygonal boundaries. As a theoretical and analytical work, the details of the novel design methodology have been provided and demonstrated by numerical models, some further worthy experimental works considering the practical conditions and environments in applications can be implemented to improve the efficiency of the heat transfer and thermal source utilizations in excited thermal techniques. The findings obtained in this paper can be used to design novel potential TO devices, including lens, source illusion, and diffusion in thermal field. Furthermore, the linear mapping function considering the changes of azimuths employed in this paper can be used to force the fluxes in the Laplace fields to efficiently transfer along certain directions in order to realize the high-gain “N-beam” diffusions [14].

## Figures and Tables

**Figure A1:**
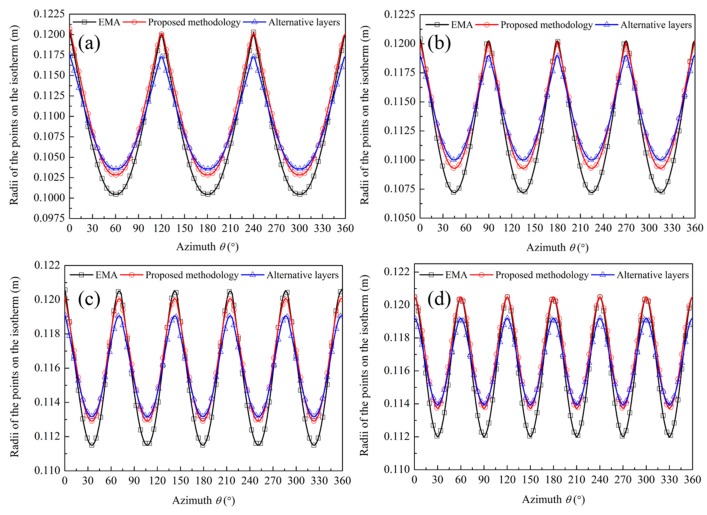
Comparison of the outside isothermal profile distributions among the ideal values of the assumed EMA in the functional regions, simulated values of the classically alternative layer configuration schemes without the reserved 10% area portions and the observed values of the proposed schemes. (**a**) The triangle scheme; (**b**) the square scheme; (**c**) the pentagon scheme; and (**d**) the hexagon scheme.

**Figure A2:**
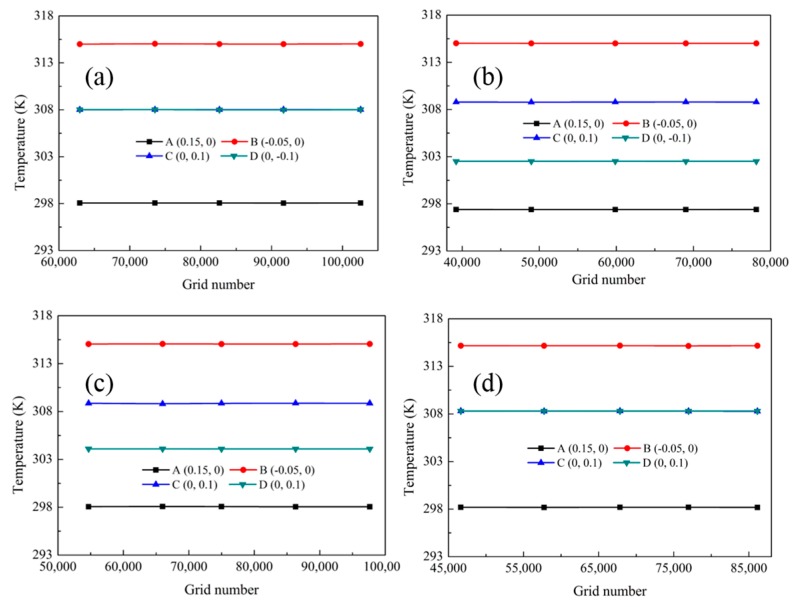
Measured temperatures of the proposed schemes with varied meshes: (**a**) the triangle scheme; (**b**) the square scheme; (**c**) the pentagon scheme; and (**d**) the hexagon scheme.

## Appendix A

In this part, the details of the employed numerical models presented in the section of “Selections of Materials and Geometric Profiles of Thermal Source Illusions” are further summarized and shown in Table A1.

**Table A1:** Summary of the detailed information of the numerical models for all the proposed schemes.

Structural Parameters (m)													
Scheme			Triangle		Square		Pentagon	Hexagon				Bare plate	
Outer radii			0.1		0.1		0.1	0.1				/	
Inner radii			0.02		0.02		0.02	0.02				/	
Background			0.4		0.4		0.4	0.4				0.4	
**Employed mediums for each proposed scheme**													
Type	I						II					Center and background	
Triangle	10% copper, 40.54% die-casting aluminum A380, 49.46% PDMS						45.05% die-casting aluminum A380, 54.95% PDMS					Nickel Steel (50% Ni)	
Square	10% copper, 41.78% ductile iron, 48.22% PDMS						49.42% ductile iron, 50.58% PDMS					Nickel Steel (50% Ni)	
Pentagon	10% copper, 42.70% steel SAE, 47.30% PDMS						50.83% steel SAE, 49.17% PDMS					Nickel Steel (50% Ni)	
Hexagon	10% aluminum alloy (6063), 39.71% steel SAE, 50.29% PDMS						44.12% steel SAE, 55.88% PDMS					Nickel Steel (50% Ni)	
Bare plate	Nickel Steel (50% Ni)												
**Material parameters**													
Material		Copper		Aluminum alloy (6063)		PDMS			Nickel steel (50% Ni)		Aluminum A380	Ductile iron	Steel SAE
Conductivity (W∙m^−1^∙K^−1^)		398		201		0.15			19.6		96.2	75	59
Density (kg∙m^−3^)		8930		2690		1000			8260		2710	7100	7830
Specific heat (J∙kg^−1^∙K^−1^)		386		900		1450			460		963	450	460
**Boundary conditions for all the schemes**													
Left wall		Right wall		Top wall		Bottom wall			Ambient	Central point source			
Thermal isolation		Thermal isolation		Thermal isolation		Thermal isolation			293 K	373 K			

## Appendix B

### Appendix B.1. Comparison between the Proposed Methodology and Well-Established Methods

In this part, we would like to make a comparison among calculated values of effective medium approximation (EMA) [9,23,43], the classically alternative layer configurations [19] without the reserved 10% area portions, and the proposed methodology, to further validate and support results. The isothermal lines of *T* = 302 K outside the devices of the above contrasts are selected to demonstrate the radial distribution characteristics of the related points with 302 K. The distributions of the isothermal lines of *T* = 302 K in each contrast are shown in Figure A1.

As can be seen from Figure A1, the radii of the points on the isotherms observed from the proposed schemes were quite approximate to the ideal values calculated by assuming EMA inside the functional regions. Furthermore, the peak values (near geometric deformations of the functional regions) of the ideal schemes and proposed schemes were close, owing to the enhanced heat transfer processes by the reserved 10% area portions. However, the valley values (near the centers of the corresponding boundaries of the functional regions) of the proposed schemes were higher than those ideal ones, due to the practical heat diffusions inside alternative medium layers. Moreover, the related values of the contrast schemes without the reserved enhancing area portions, i.e., employing the classical medium configurations [26,27,28,29,30,31,32,33,34], were also presented above. As it indicates, the classical medium configuration scheme would also perform the approximate characteristics to the ideal schemes. However, such approximations were weaker than the proposed schemes, as the peak values could not approach the ideal ones, and the valley values were higher than those of the proposed schemes. That is, the thermal energy inside the central medium cells diffused faster than that in the cells near the geometric deformations, due to the smaller heat transfer distances of the central cells. Hence, the profiles of the isotherms *T* = 302 K of the proposed schemes would be more approximate to the ideal EMA ones, compared with the classical medium configuration schemes without the reserved thermal enhancing area portions.

### Appendix B.2. Independence Analysis of the Presented Schemes

The independence analysis of the presented schemes based on the proposed methodology is operated in this part. In order to complete the independency analysis, four measured points, i.e., A (0.15, 0), B (−0.05, 0), C (0, 0.1), and D (0, −0.1), are selected in all the schemes. Furthermore, five types of meshes with different grid numbers were selected for each mentioned scheme and are shown in Table A2.

**Table A2:** Different meshes for each original scheme.

Illusive Schemes (Figure 6)	Grid Number				
	1	2	3	4	5
Triangle	39,214	48,961	59,865	69,023	78,165
Square	46,650	57,732	67,836	76,962	86,142
Pentagon	54,685	65,985	74,986	86,316	97,645
Hexagon	62,947	73,546	82,643	91,687	102,567

The values of the five measured points in each scheme with different meshes are illustrated in Figure A2. It can be seen that the temperatures of each measured point had little fluctuations with the increasing grid numbers. That is, the observed results for the schemes were independent of the meshes, i.e., the results are accurate to support the novelties.
